# An optimized detection model for micro-terrain around transmission lines

**DOI:** 10.1038/s41598-025-88385-7

**Published:** 2025-02-11

**Authors:** Feng Yi, Chunchun Hu

**Affiliations:** School of Geodesy and Geomatics, Wuhan University, Wuhan, 430079 China

**Keywords:** DEM (Digital elevation model), Transmission lines, Parallel random forest, Micro-terrain, Characteristic factors, Geology, Geomorphology

## Abstract

Detecting micro-terrain is essential for the effective layout and maintenance of transmission lines. To address the issues of detection incompleteness, classification ambiguity, and inefficiency in traditional methods, particularly the challenge of distinguishing between saddle and canyon micro-terrain, this paper optimizes the calculation of micro-terrain features and the strategy of micro-terrain detection, and explores a detection method of micro-terrain around transmission lines based on the GPU parallel random forest. This paper employs the GPU parallel random forest model as the extraction framework, leveraging the computational speed advantage of GPU parallel technology for handling large datasets and the robustness inherent in the ensemble approach of random forests. The DEM data of 49 transmission lines in the study area was used for micro-terrain detection experiments. Most of these 49 routes are situated in mountainous regions with complex terrain and contain diverse micro-terrain categories along their paths, rendering them highly representative. The experimental results demonstrate that the proposed method effectively identifies atypical micro-terrain types and four typical micro-terrain types—saddle, canyon, alpine watershed, and uplift—with a classification accuracy of 97.96% and a Kappa coefficient of 0.974. Compared to the traditional method, which achieves a classification accuracy of 75.19% and a Kappa coefficient of 0.642, the proposed method demonstrates a clear improvement in performance. Moreover, by employing the parallel model, the acceleration ratios for training and classification reach 50.57 and 109.06, respectively, significantly improving the efficiency of micro-terrain detection for large-scale regions. These findings could significantly enhance transmission line maintenance and layout planning by providing more accurate micro-terrain data, enabling better decision-making and resource allocation for infrastructure development and disaster risk mitigation.

## Introduction

**Fig. 1:**
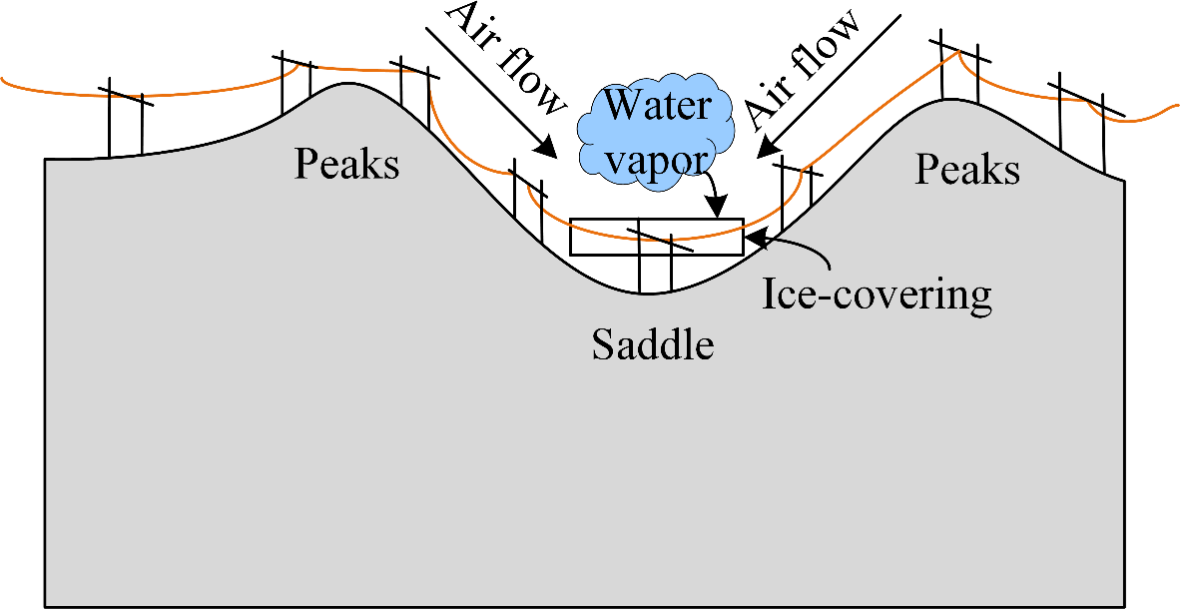
Local meteorological changes caused by the saddle.

Recently, China’s electricity demand has experienced rapid growth, with projections indicating that total electricity consumption for the entire society will reach 9.4 trillion to 9.8 trillion kW·h by 2025^1^. In response, China’s power grid is continuously expanding and evolving to enhance transmission capacity. Certain transmission lines pass through regions characterized by complex terrain and severe weather conditions making them vulnerable to damage from meteorological events such as ice-covering, strong winds, and lightning, leading to significant economic losses^2^. Fig. 1 illustrates the local meteorological changes like strong winds and water vapor augment caused by the saddle surrounding the transmission lines. As the airflow converges and accelerates in the low-lying regions of the saddle, wind speed intensifies, leading to higher wind pressure and intensified line vibration. During winter, the accumulation of water vapor in the saddle area frequently leads to ice-covering disasters on the transmission lines. Among these prevalent disasters, ice-covering poses a particularly severe threat to transmission lines. Ice-covering imposes additional loads on transmission line components, including wires and insulators, leading to faults like wire breakage and insulator breakdown, thus presenting a serious threat to the safe operation of the power grid^3^. A globally recognized example of transmission line damage caused by ice storms is the 1998 event in Eastern Canada and the Northeastern United States^4^. The primary cause of ice-covering disasters is the condensation of the water vapor near transmission lines, which results in the formation of ice layers on the conductors. This process is largely influenced by micro-terrain, as complex micro-terrain can change air humidity, airflow direction, and speed, which, in turn, affects the formation of ice accumulation^5^.

Micro-terrain refers to areas with a smaller scale of analysis and more complex terrain than macro-terrain. The micro-terrain surrounding transmission lines encompasses various categories. For a more detailed definition and description of these categories, please refer to Sect. “Micro-terrain categories”. By analyzing the micro-terrain surrounding transmission lines, the potential locations vulnerable to meteorological disasters can be identified during the construction phase, thereby minimizing damage to transmission lines and ensuring the reliable and uninterrupted operation of the power transportation system.

In the field of terrain recognition, researchers have developed various methods and models for automatic terrain classification. These methods can be broadly categorized into three types based on their characteristics: clustering-based methods, rule-based methods, and typical sample point-based methods^6^. Clustering-based methods, such as K-means and hierarchical clustering, classify terrain by grouping similar data points. These methods are particularly suitable when prior knowledge is unavailable. While they offer a high degree of automation, they often struggle to effectively capture complex terrain features, resulting in classifications that may lack clear geological significance^7^. Rule-based methods, on the other hand, rely on expert knowledge to define classification rules^8^. While they are highly interpretable, their lack of flexibility limits their effectiveness in handling complex or dynamic terrain features. Typical sample point-based methods utilize labeled samples and employ supervised learning techniques, such as machine learning, to train models for terrain classification^9,10^. These methods generally achieve high classification accuracy when sufficient labeled data is available. However, their performance can be hindered by limitations in sample quality and quantity, and they often face challenges related to high computational complexity^6^. With the advent of deep learning, semantic segmentation methods have also been applied to terrain classification^11,12^. These approaches collectively provide valuable insights for the research on micro-terrain extraction of transmission lines presented in this paper.

Research on transmission lines micro-terrain has a long-standing history. Initial researches primarily explored the relationship between transmission lines micro-terrain and meteorological disasters. Koval et al.^13^ examined the impact of extreme weather on transmission lines by analyzing an Alberta provincial utility’s transmission line outage database and Alberta Environmental Service weather databases over a 20 year period. Their analysis highlighted the critical role of geographical location in transmission line vulnerability. Drawing on instances of ice-covering disasters in Yunnan Province, Zhang et al.^14^ introduced the concepts of micro-terrain and micro-meteorology, investigating their influence on ice-covering mechanisms. Their study proposed technical strategies for the operation and maintenance of transmission lines. However, these studies largely relied on qualitative theoretical analyses, offering limited accurate and specific data references for transmission line operation and maintenance. Quantitative classification results, in contrast, often provide more actionable insights. Drăguţ et al.^15^ presented an object-based approach for automatic terrain classification using SRTM(shuttle radar topography mission) data. This method relied on two terrain characteristics extracted from DEM: elevation and standard deviation of elevation. As DEM data acquisition advanced, quantitative analyses of micro-terrain became increasingly feasible. Jing et al.^16^ proposed a method for extracting transmission line saddles integrated terrain feature lines and characteristic factors, successfully correlating saddle locations with areas of severe ice-covering hazards. However, the process for extracting feature lines was complex and unsuitable for widespread application. To improve identification efficiency, Zhou et al.^17^ introduced a micro-terrain extraction technique based on a decision table, identifying five micro-terrain types. Nevertheless, this method exhibits suboptimal performance in distinguishing between the saddle and the canyon micro-terrain due to their potential confusion. In general, the methods employed in the aforementioned studies are relatively traditional and heavily reliant on rule-based knowledge. Additionally, these approaches are often inefficient in micro-terrain extraction due to the complexity of overlay analysis operations. These limitations underscore the inadequacy of existing methods in meeting the demands of large-scale datasets and high-precision classification. To overcome these issues, there is a pressing need for methods capable of efficiently processing large-scale terrain data and extracting micro-terrain features using optimized algorithms.

To overcome these issues, research on machine learning-based micro-terrain detection has gained traction. For instance, Dornik et al.^18^ utilized SRTM data and employed a random forest classifier to meticulously classify and extract various mountain terrain types, enabling the fine-scale automatic extraction of micro-terrain. Wang et al.^19^ computed weighted characteristic factors using a random forest model, reducing the influence of subjectivity in factor selection and improving slope classification accuracy. However, this study did not address enhancements to the model’s time performance. Similarly, Zhou et al.^20^ utilized a convolutional neural network model for the classification of mountain micro-terrain, successfully identifying six types of mountain micro-terrain. While this approach mitigated the issue of classification incompleteness inherent in traditional methods, the model’s scalability remained a challenge. Current research on machine learning-based micro-terrain detection primarily focuses on improving classification accuracy, with limited attention given to classification efficiency. Conventional machine learning methods often require significant time when processing high-resolution DEM data. Enhancing the efficiency of micro-terrain detection in the context of large-scale high-resolution data remains a significant challenge. The GPU parallel random model proposed in this paper leverages GPU acceleration technology to overcome the time performance limitations often overlooked by conventional machine learning methods in terrain classification. Related article^21^ demonstrates that terrain factor calculations performed in a CUDA environment can significantly improve computational efficiency compared to traditional serial processing. The findings of the related article indicate that, for large grid scales, the maximum speedup achieved can reach 24.39^21^, underscoring the feasibility of applying GPU acceleration to micro-terrain extraction.

To break through the limitations of traditional methods, this study focuses on optimizing the calculation of micro-terrain features and refining the extraction strategy to overcome issues such as incomplete and ambiguous classifications. Notably, it tackles the challenge of distinguishing between the saddle type and the canyon type. To address the inefficiency of conventional machine learning methods, a GPU-parallel random forest-based micro-terrain extraction method for power transmission lines is proposed. Experimental results demonstrate that the proposed method improves classification accuracy by 22.77% and Kappa coefficient by 0.332 compared to traditional approaches, showcasing significant advancements. Furthermore, the method also exhibits excellent performance in terms of time efficiency. This study also explores potential limitations, such as model scalability and computing resource requirements.

In the context of transmission line planning and design, it is crucial to consider the distribution characteristics of micro-terrain along the line to optimize layout, reduce engineering costs, and minimize environmental impact. The findings of this research provide precise micro-terrain classification data to support line site selection and enable the prediction of potential disaster risks along the route. For instance, by integrating micro-terrain extraction results with meteorological data, a model can be developed to identify areas prone to ice-related disasters, offering a reliable foundation for disaster prevention. Additionally, the technical approaches presented in this study offer valuable support for the rapid and large-scale extraction of micro-terrain, broadening their applicability to other relevant domains.

## Methodology

### Micro-terrain categories

**Fig. 2:**
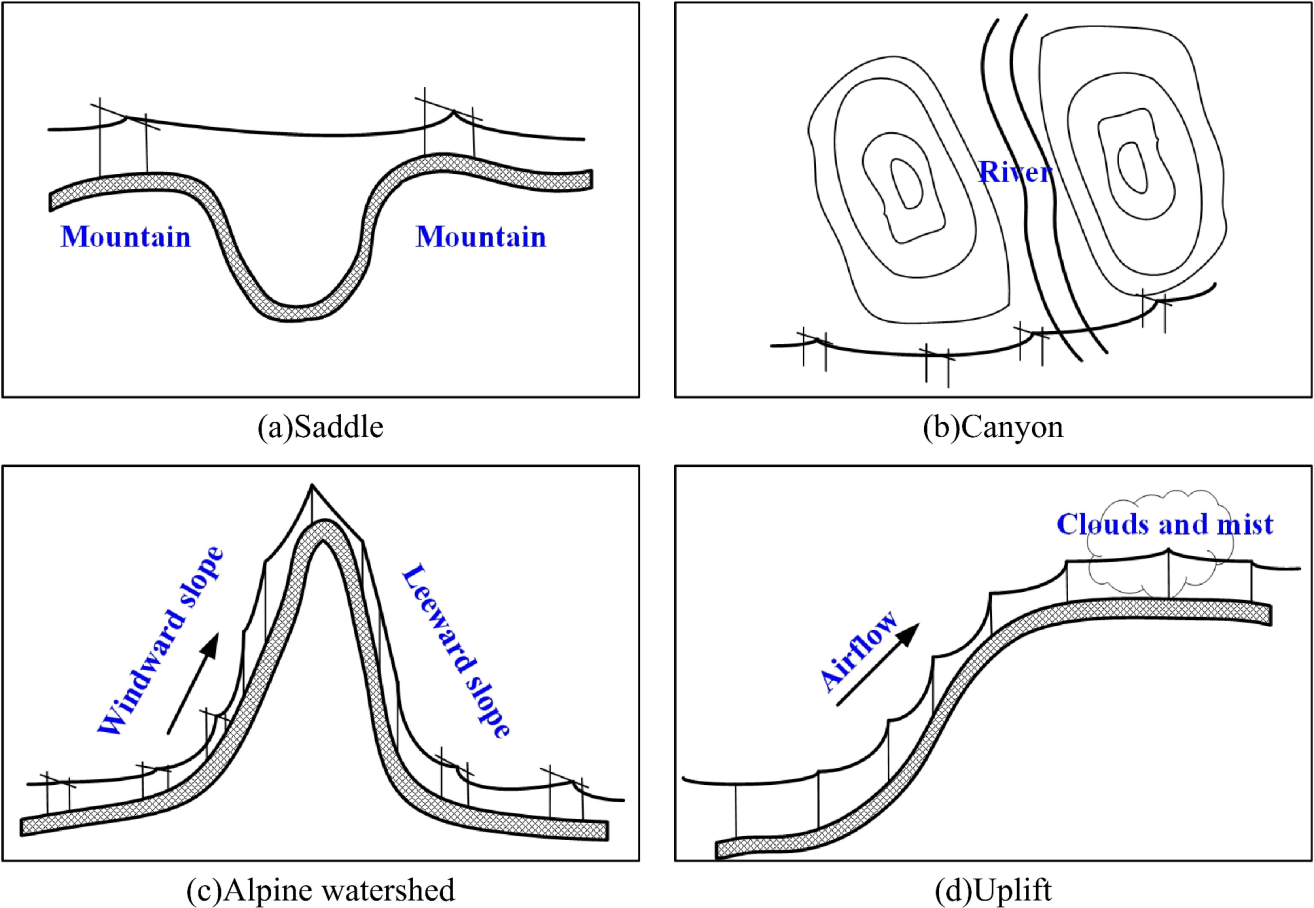
Delineation of typical micro-terrain types.

Figure 2 illustrates the typical micro-terrain types around transmission lines, including saddles, canyons, alpine watersheds, and uplifts^17^. When the transmission lines intersect such micro-terrain, they become highly susceptible to meteorological disasters. Take the uplift type (Fig. 2d) as an example: cold airflow ascends along the hillside, resulting in the formation of clouds and mist on the top or terrace. During the winter cold waves, this phenomenon makes transmission lines particularly vulnerable to severe ice-covering disasters.

The saddle (Fig. 2a) refers to a narrow passage or depression between two peaks, often representing the lowest or relatively low-lying section between them. The canyon (Fig. 2b) refers to a narrow fissure or gully created by surface water erosion, typically formed over time by the erosive action of rivers on rock formations. Although the definitions of these two micro-terrain types differ, their geographical structures are relatively similar. Both exhibit low-lying characteristics, making it challenging to differentiate between the saddle and the canyon. The alpine watershed (Fig. 2c) refers to the mountain ridge or highland that separates two adjacent river basins. When the line crosses the watershed, strong winds or ice cover are likely to occur. The uplift type (Fig. 2d) refers to the sudden peaks rising from the ground in plains or hills, or the platforms and cliffs in the basin that are lower on one side and higher on the other. For a detailed definition of micro-terrain categories and an analysis of specific cases, please refer to the paper proposed by Zeng^22^, which discussed them in more detail.

Related studies have demonstrated a strong correlation between the micro-terrain of transmission lines and the natural disasters impacting these lines. In previous study^17^, the authors compared the micro-terrain extraction results from nine transmission lines with disaster-prone areas along these routes. Their analysis revealed that over 90% of the micro-terrain for all the lines was situated in disaster-prone sections, highlighting the critical role of micro-terrain in influencing the vulnerability of transmission lines to natural disasters.

Micro-terrain surrounding transmission lines is typically categorized based on mountainous regions. However, certain lines pass through non-mountainous areas like residential zones and farmlands. To improve the adaptability of detection method of the micro-terrain, areas not considered typical micro-terrain are now uniformly labeled as “atypical micro-terrain type”. In our research, the sample dataset for experiments will consist of five category labels: four representing typical micro-terrain and one for atypical micro-terrain type.

### Optimized computation of characteristic factors

In micro-terrain detection, characteristic factors are parameters employed to describe the surface morphology and information. Commonly utilized characteristic factors comprise slope, TPI (Topographic Position Index)^23^, terrain undulation, and curvature. To facilitate a better understanding of these characteristic factors, we have compiled a glossary describing them, as presented in Table 1.

**Table 1:** Definition of terrain factors mentioned in this article.

Factors types	Definition
Slope	The angle between the terrain surface and the horizontal plane, used to describe the slope of the terrain
TPI	A parameter that describes the height of a point relative to the surrounding terrain
Undulation	A parameter that describes the range of terrain elevation variation within a certain area, usually calculated by the difference between the maximum and minimum elevations in the area.
Profile curvature	Characterizes the curvature of the terrain surface along the slope direction. In differential geometry, it can be calculated based on the second-order derivative of the elevation function

The calculation of slope and profile curvature is rooted in the principles of differential geometry. To enhance readers’ understanding of these concepts, slope will be used as an example for detailed explanation. In differential geometry, the slope is determined by the magnitude of the gradient vector $\:\nabla\:f(x,y)$ of the elevation function $\:z=f(x,y)$. Consequently, the slope $\:S$ at a given point on the surface can be computed using the following formula^24^:

1 $$\:S=arctan\sqrt{{\left(\frac{\partial\:f}{\partial\:x}\right)}^{2}+{\left(\frac{\partial\:f}{\partial\:y}\right)}^{2}}$$

Where the term $\:\partial\:f/\partial\:x$ denotes the partial derivative of the elevation value along the north-south direction, while $\:\partial\:f/\partial\:y$ represents the partial derivative along the east-west direction.

To effectively identify micro-terrain, we optimize the computational methods of some terrain factors. The optimization of TPI primarily concentrates on determining the optimal analysis window. Profile curvature indicates the degree of curvature of the terrain morphology, with adaptive neighborhood methods enhancing its computational accuracy. These optimizations are aimed at capturing more accurate terrain features. Smaller windows capture finer details, such as local terrain variations, while larger windows offer a broader perspective, helping to identify overall landscape patterns. The window size determined through these optimization methods strikes a balance between detailed analysis and the broader terrain context, leading to more precise terrain feature extraction.

#### Optimized TPI

This study optimizes TPI computation to determine the optimal calculation window. The TPI commonly used to evaluate the spatial relationship between a location and its surrounding topography. The calculation formula is as given follows:

2 $$\:TPI={z}_{i}-\stackrel{-}{z}$$

where $\:{z}_{i}$ is the elevation value of point $\:i$, $\:\stackrel{-}{z}$ is the average value of all elevation points within the neighborhood of point $\:i$.

**Fig. 3:**
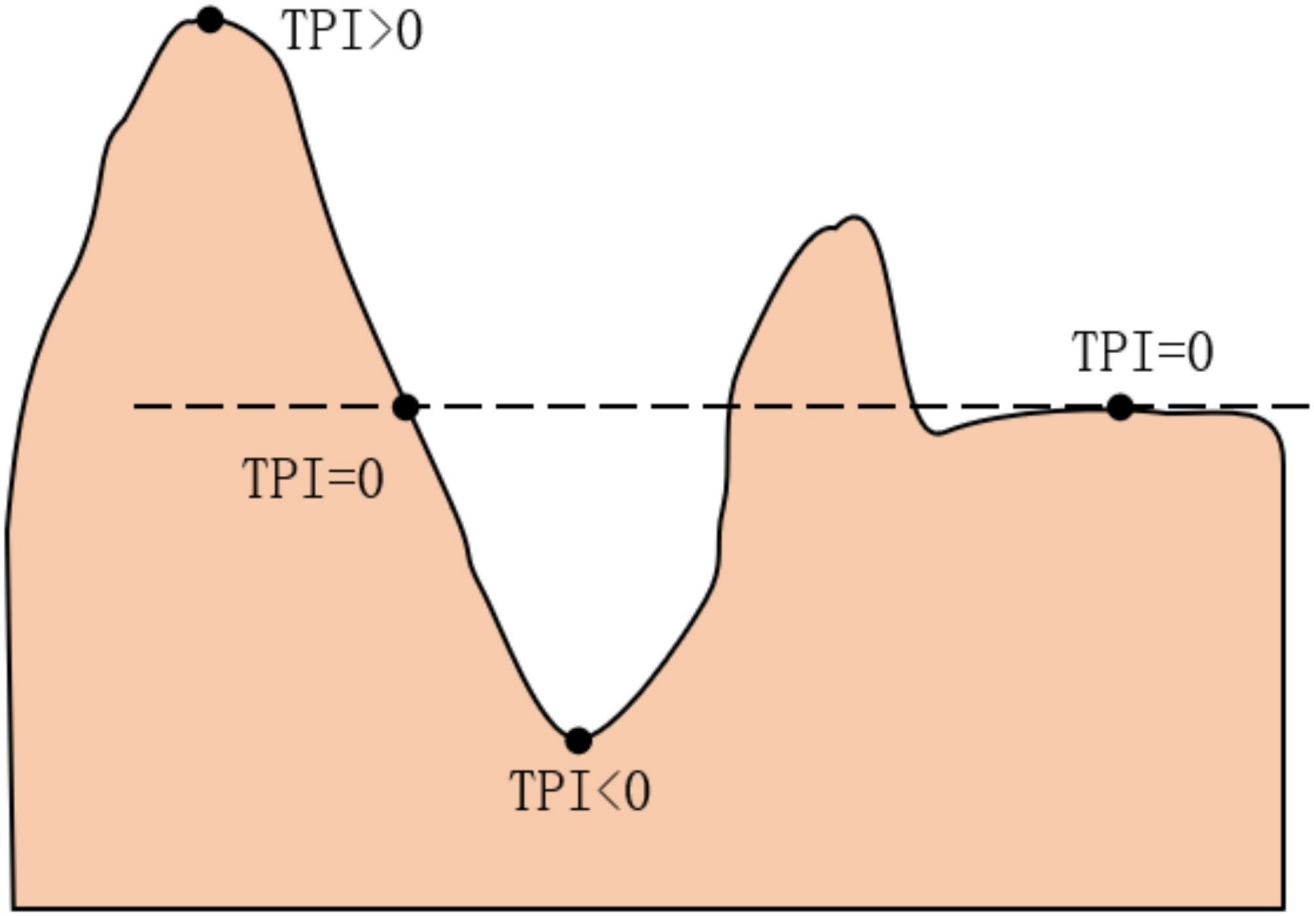
TPI values for different terrains.

TPI effectively illustrates the terrain’s undulations, as demonstrated in Fig. 3, making it a widely used tool for micro-terrain detection. However, computing TPI using formula (2) presents challenges in determining the classification threshold. Typically, normalizing TPI is required before its application^23^. Generally, TPI is computed twice—once with a small calculation window and once with a large one— resulting in both small and large TPI values. This method, known as “dual TPI combination”, was utilized by Zhou et al.^17^ to detect micro-terrain.

**Fig. 4:**
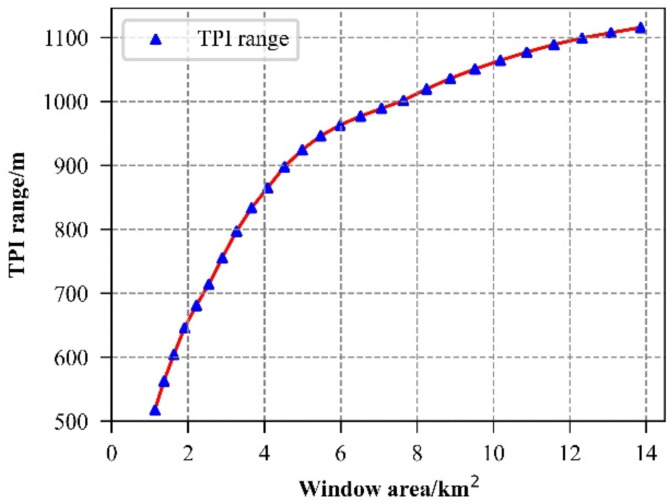
TPI range at different windows.

The TPI values vary depending on the window size, which presents a challenge in selecting the optimal window for TPI calculation. For instance, to determine the optimal window for computing large TPI, we calculate the range of TPI (the difference between the maximum and minimum TPI values in the study area for a specific calculation window) .According to Zhou et al.’s article^17^, the TPI remains effective in capturing detailed micro-terrain features when the radius exceeds 600 m, making it suitable for describing local terrain changes. However, when the window radius exceeds 2000 m, TPI gradually loses sensitivity to elevation changes, thereby neglecting the finer details of the micro-terrain. Therefore, we aim to identify the optimal window size for computing large TPI by testing radii from 20 to 70 pixels in 2-pixel increments (i.e., 20, 22, 24, 26, …, 70), based on the DEM raster image resolution of 30 m. Analysis of Fig. 4 reveals a knee point in the change process. Prior to the knee point, the TPI range experiences rapid growth with increasing window size, followed by a slowdown in the rate of increase. This shift is attributed to the increased stability of the mean elevation benchmark at each point within the study area at the knee point. At this stage, the mean elevation of each point can better reflect its relative position, resulting in a more accurate TPI value^25^. Therefore, the window corresponding to the knee point is considered the optimal calculation window. Figure 4 illustrates that the area of the optimal calculation window for large TPI is approximately 6 km^2^.

In our research, considering the uncertainty of subjective factors in traditional visual determination method, we employ the mean variable-point analysis method^26^ to determine the knee point position for TPI calculation. The calculation process is outlined as follows:

1. Divide the TPI range by the corresponding window area to obtain the TPI range per unit area, and take the logarithm to obtain the data sequence $\:\left\{{x}_{i}\right\}$, $\:\:i=$1, 2, 3 ,… ,$\:N$,$\:\:N$ is the number of samples. According to formula (3), the variance of the entire data sequence is calculated:

3 $$\:S=\sum\:_{i=1}^{N}{\left({x}_{i}-\stackrel{-}{x}\right)}^{2}$$

where $\:\stackrel{-}{x}$ is the average value of $\:\left\{{x}_{i}\right\}$.

(2) Successively, select $\:{x}_{i}$($\:i=$2,3,…,$\:N$) as the cutoff point to divide the sequence into two subsequences $\:{x}_{{t}_{1}}=\left\{{x}_{1},{x}_{2},\dots\:,{x}_{i-1}\right\}$ and $\:{x}_{{t}_{2}}=\left\{{x}_{i},{x}_{i+1},\dots\:,{x}_{N}\right\}$, and calculate the sum of the variances $\:{S}_{i}$ of the two subsequences according to formula (4):

4 $$\:{S}_{i}=\sum\:_{{t}_{1}=1}^{i-1}{\left({x}_{{t}_{1}}-{\stackrel{-}{x}}_{i1}\right)}^{2}+\sum\:_{{t}_{2}=i}^{N}{\left({x}_{{t}_{2}}-{\stackrel{-}{x}}_{i2}\right)}^{2}$$

where $\:\:{\stackrel{-}{x}}_{i1}$and $\:{\stackrel{-}{x}}_{i2}$ are the arithmetic means of the subsequences $\:{x}_{{t}_{1}}$ and $\:{x}_{{t}_{2}}$ respectively.

(3) Calculate the difference between $\:S$ and $\:{S}_{i}$ in turn to get the result sequence $\:\{S-{S}_{i}\}$. The truncation point $\:{x}_{i}$ corresponding to the maximum value in the result sequence is the target knee point.

**Fig. 5:**
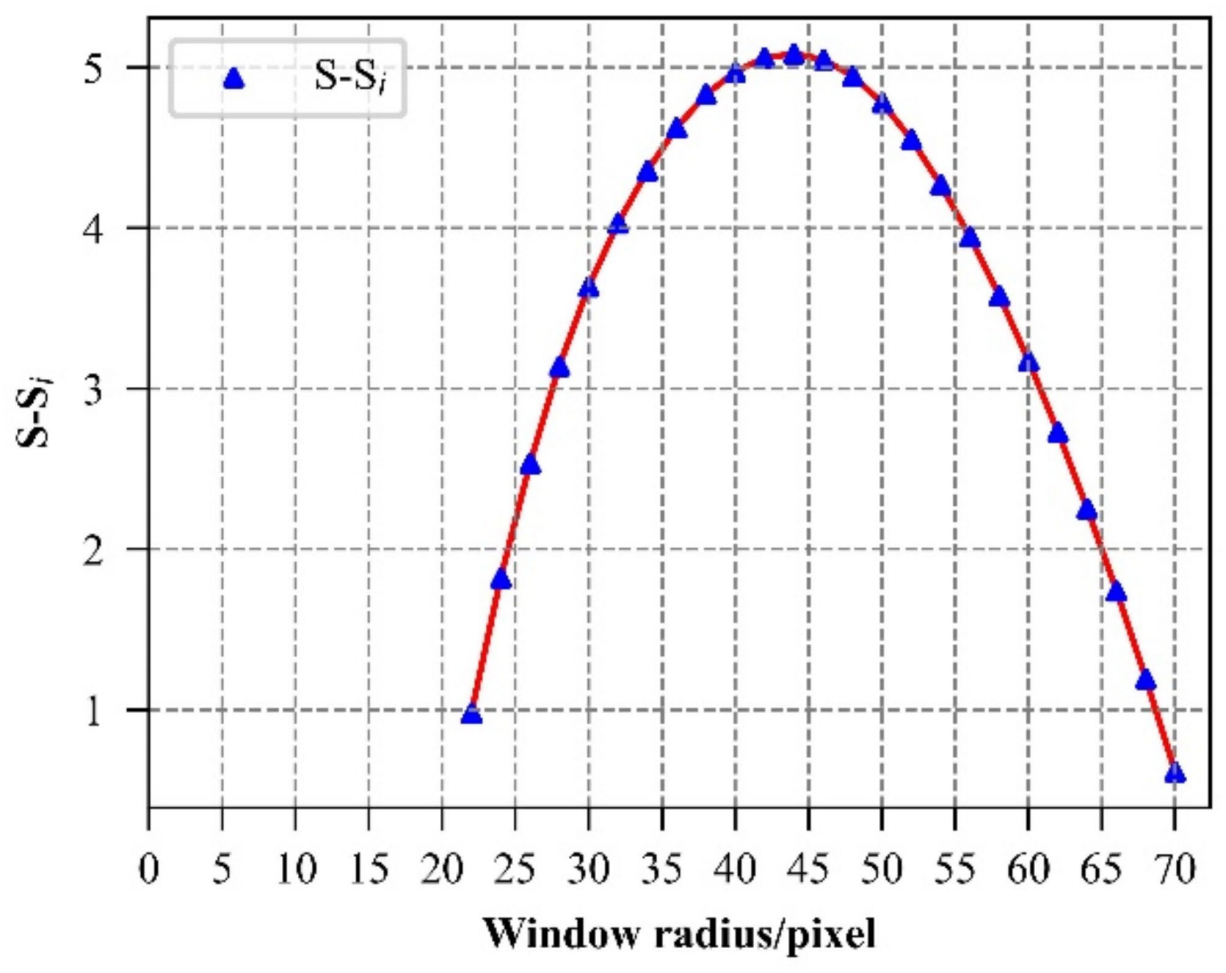
Change curve of difference between $\:S$ and $\:{S}_{i}$.

Fig. 5 depicts the curve showing the difference between $\:S$ and $\:{S}_{i}$, derived from the TPI data within the study area. From Fig. 5, it is evident that the optimal calculation window radius for large TPI is 44 pixels, corresponding to a window area of 5.47 km^2^.

#### Profile curvature calculation based on the adaptive neighborhood

Given that profile curvature effectively captures the terrain morphology’s curvature, it is extensively applied in landslide detection^27^, soil and water erosion analysis^28^, and other related fields. However, using a fixed window to calculate profile curvature may not accurately capture micro-terrain details. The adaptive neighborhood method dynamically adjusts the calculation window size based on local features, allowing for a more precise representation of micro-terrain details and improving the accuracy of characteristic factors. Unlike the fixed-size window, the adaptive neighborhood method employs smaller windows for areas requiring higher precision and larger windows for flatter terrain, thereby balancing efficiency and accuracy.

This paper employs an adaptive neighborhood approach to compute profile curvature. The minimum neighborhood window size for calculating profile curvature is typically set to 3 × 3 pixels. To meet the accuracy requirements of micro-terrain analysis, the maximum window size is limited to 500 × 500 m, as larger windows risk excessive smoothing, which could obscure critical terrain details. The deviation (DEV) of the average elevation is calculated sequentially for neighborhood windows ranging from 15 × 15 pixels to 3 × 3 pixels, in descending order. DEV serves as an indicator for assessing terrain complexity within the window^29^. The calculation formula is as follows:

5 $$\:\left\{\begin{array}{c}DEV=\frac{{z}_{0}-\stackrel{-}{z}}{SD}\\\:SD=\sqrt{\frac{1}{n-1}\sum\:_{i=1}^{n}{\left({z}_{i}-\stackrel{-}{z}\right)}^{2}}\end{array}\right.$$

where $\:{z}_{0}$ is the elevation value of a point; $\:\stackrel{-}{z}$ is the mean value of elevation in the neighborhood; $\:SD$ is the standard deviation of elevation in the neighborhood; $\:n$ is the number of points in the neighborhood; and $\:{z}_{i}$ is the raster points in the neighborhood.

DEV changes according to the window size. Once DEV reaches the threshold, the corresponding window becomes the adaptive neighborhood window for calculating profile curvature at the specific point. In this study, the preset threshold is set to 0.1, which is determined based on experimental data and empirical data.

### Optimized strategy for detecting micro-terrain

Due to the geographical similarity between saddles and canyons, solely utilizing the “dual TPI combination”^17^ method fails to fully differentiate between them. While TPI describes a point’s relative position within its surrounding terrain, profile curvature offers a more precise representation of the morphological attributes and structural characteristics of the local surface. To enhance the capture of terrain characteristics on small-scale surfaces, this study incorporates profile curvature as a micro-terrain characteristic factor, replacing small TPI.

The theoretical feasibility of substituting small TPI with profile curvature was assessed using the Pearson correlation coefficient method. Table 2 presents the correlation analysis results of several characteristic factors, showing a strong a correlation coefficient of 0.879 between profile curvature and small TPI, which suggests that profile curvature can theoretically replace small TPI as a characteristic factor.

**Table 2:** Correlation analysis results of some characteristic factors.

Characteristic factor	Profile curvature	Large TPI	Small TPI
Profile curvature	1	0.690	0.879
Large TPI	0.690	1	0.647
Small TPI	0.879	0.647	1

To further evaluate the impact of profile curvature, we adopted the classification decision scheme outlined by Zhou et al.^17^. By substituting profile curvature for small TPI and incorporating the elevation coefficient of variation, we refined the characteristic factors combination. Additionally, selecting an appropriate classification threshold is critical for optimizing the classification strategy. For instance, while standardized TPI often uses − 1 and 1 as classification thresholds, our experiments indicate that thresholds of -0.8 and 0.8 yield more accurate outcomes for micro-terrain detection in this study. After iterative adjustments, we finalized an optimized decision table for identifying saddles and canyons (see Table 3). Using this decision table, sample points representing saddles and canyons were extracted for further analysis. (The unit of profile curvature in this paper is the reciprocal of the x and y units in the output coordinate system.)

**Table 3:** Decision table for detection of saddle and canyon micro-terrain.

Category	Characteristic factor		
	Optimal large TPI	Profile curvature	Elevation coefficient of variation
Saddle	>-0.8	<-0.000 3	\
	<-0.8	<-0.000 3	< 0.04
Canyon	<-0.8	<-0.000 3	> 0.04

In addition to the saddle and canyon types, this study incorporates optimal large TPI, slope, undulation, and profile curvature to form a comprehensive set of characteristic factors for detecting various other micro-terrain types. Likewise, after numerous adjustments to the classification thresholds, the detection decision table (see Table 4) has been finalized.

Adjusting the threshold is a critical step in optimizing the performance of micro-terrain detection. Initially, we conducted a preliminary analysis of the performance of these terrain factors in different micro-terrain extractions to determine the potential threshold ranges. Subsequently, classification results were obtained based on the initial thresholds, and these were iteratively refined and adjusted according to the classification results during experiments. Particular attention was given to the influence of terrain factor categories and specific segmentation thresholds. For instance, in determining the classification thresholds for the alpine watershed in Table 4, it was observed that variations in slope had minimal impact on distinguishing between it and other types. As a result, no specific slope threshold was defined for this category. Conversely, for the uplift type in Table 4, it was found that this type was distributed within the range of TPI between − 0.8 and 0.8 and greater than 0.8. Hence, a finer division was implemented to ensure the classification results aligned more closely with the actual distribution of micro-terrain.

**Table 4:** Decision table for detection of other micro-terrain types.

Category	Characteristic factor			
	Optimal large TPI	Profile curvature	Slope/(°)	Undulation /m
Alpine watershed	> 0.8	> 0.000 3	\	> 450
Uplift	> 0.8	> 0.000 3	\	< 450
	−0.8～0.8	\	> 15	> 300
Atypical type	Micro-terrain beyond typical micro-terrain types			

### Parallel random forest model for micro-terrain detection

Random forest is an ensemble learning algorithm consisting of multiple decision trees, each acting as weak classifiers. It combines the prediction outcomes of these trees through voting or averaging, ultimately resulting in the final classification or regression. The algorithm’s core principle involves constructing multiple decision trees by randomly selecting samples and feature subsets. Specifically, “bootstrap sampling” is employed to generate a dataset for each tree by randomly selecting samples from the original dataset with replacement. Instead of using all features, a random subset of features is selected for training each tree. Random forest offers several advantages, including robust processing capabilities for high-dimensional data, resistance to overfitting, and improved robustness^30^.

CUDA (Compute Unified Device Architecture) is a versatile parallel computing platform and programming model developed by NVIDIA. It enables developers to harness NVIDIA’s GPUs for highly efficient, parallel computations^31^. The CUDA kernel function can initiate numerous threads on the GPU to execute the same operation concurrently in parallel mode. These threads are structured into blocks and grids to leverage the multiple Streaming Processors in the GPU, enabling simultaneous data processing for high-performance parallel computing^31^.

**Fig. 6:**
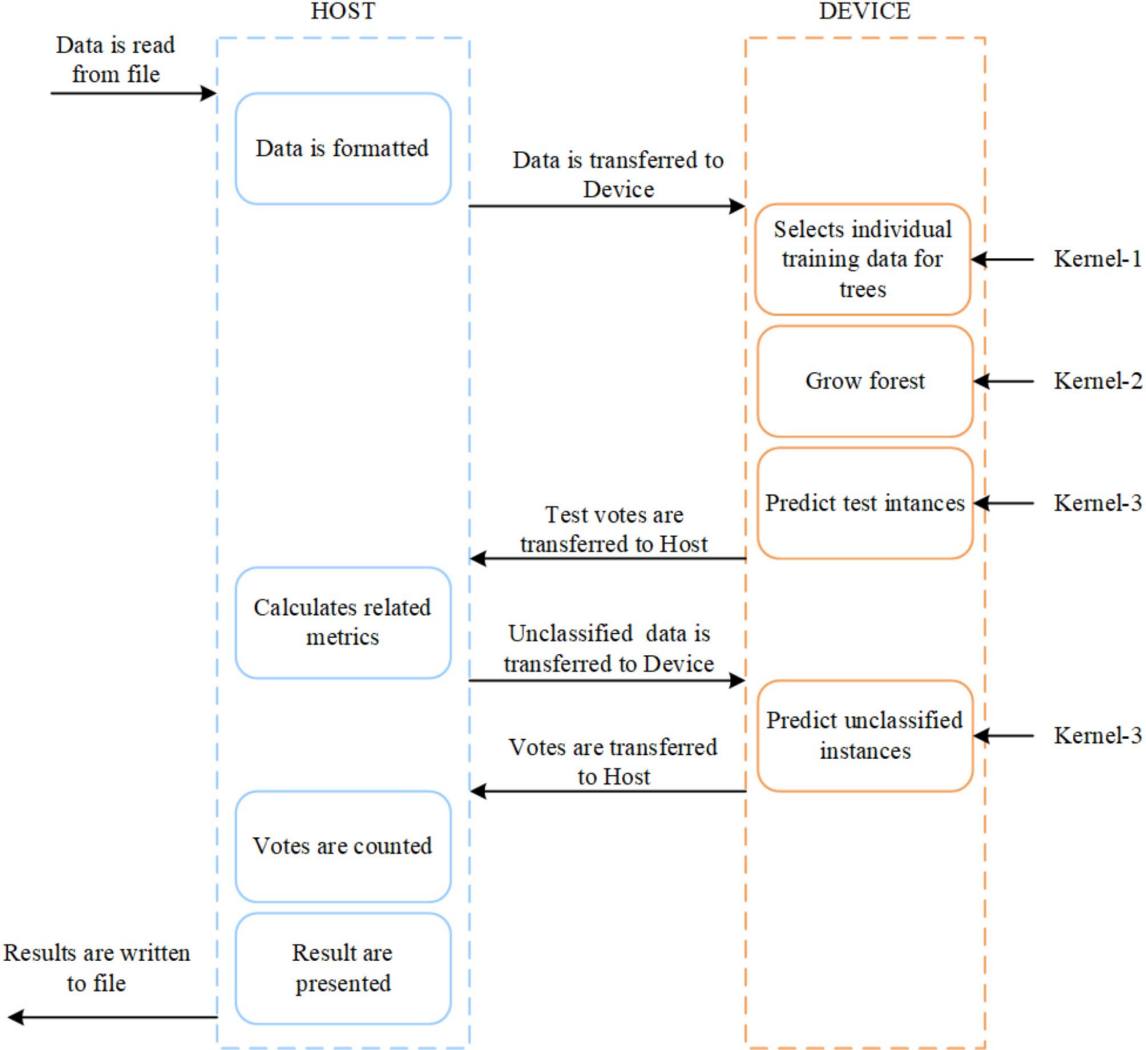
Random forest CUDA parallelization process.

In this paper, the random forest employs three CUDA kernel functions (see Fig. 6) for parallel micro-terrain detection. Kernel-1 function parallelizes the bootstrap sampling process, allocating characteristic factor data and category labels necessary for training to individual decision trees. Kernel-2 function facilitates parallel training of decision trees, with each tree being constructed simultaneously by its respective threads. Kernel-3 parallelizes prediction, allocating a thread to each unclassified point to perform parallel prediction of the category labels^32,33^. Micro-terrain detection typically involves high-resolution DEM raster over extensive areas, resulting in substantial data volumes. Constructing a random forest model for micro-terrain detection exclusively on the CPU is time-intensive. Alternatively, leveraging the GPU for parallel construction can significantly improve efficiency.

While CUDA technology provides significant advantages in parallel computing, implementing parallel random forest models poses challenges, such as efficiently transferring data to the GPU when processing large datasets. Additionally, computational bottlenecks, including memory bandwidth limitations or inefficient thread scheduling, can degrade GPU performance. Therefore, optimizing data transfer, memory usage, and thread management are critical for developing efficient parallel random forest models.

## Experimental results and discussion

**Fig. 7:**
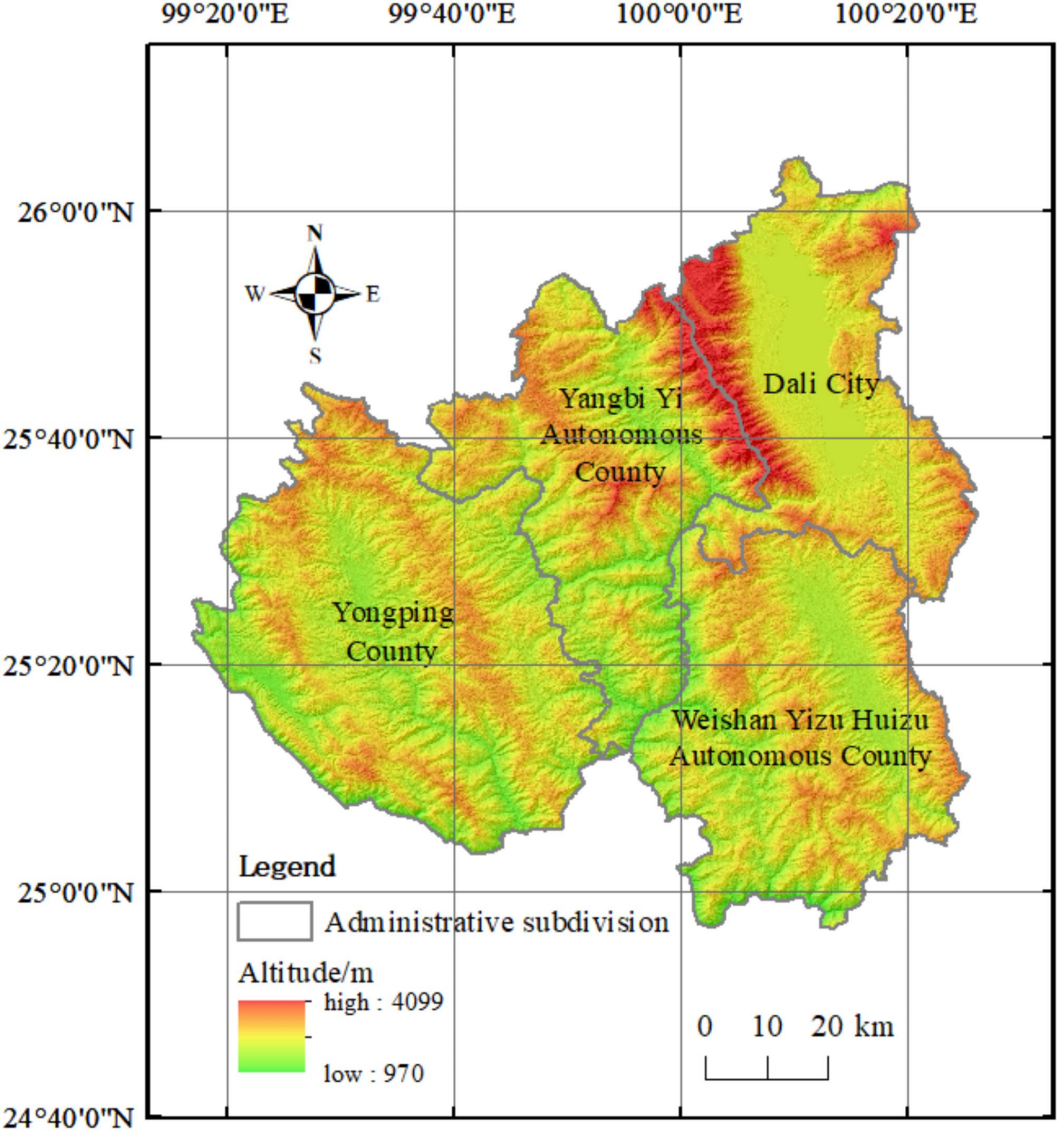
Overview of the study area.

The chosen transmission lines are situated within four counties or cities under the jurisdiction of Dali Bai Autonomous Prefecture. Figure 7 depicts the overview of the study area. The study area lies at the intersection of the Yunnan-Guizhou Plateau and the Hengduan Mountains, spanning an elevation range from 970 to 4099 m. The experiments utilized DEM raster data with a resolution of 30 m as the primary data source.

Based on Tables 3 and 4, and prior knowledge, the most representative points within the study area are extracted to form the sample dataset. To ensure sample balance, each category was downsampled to 8192 samples, resulting in a total of 40,960 sample points. The training set comprises 70% of the sample dataset, with the remaining 30% allocated to the test set.

To ensure the effectiveness of the research methodology, this study implemented a comprehensive experimental process encompassing data collection, preprocessing, model training, and result analysis. During the data preprocessing stage, we performed denoising and georeferencing on the raw data to eliminate extraneous interference. Subsequently, micro-terrain features were extracted using the optimization method proposed in this paper. These extracted features were then combined with the strategy table mentioned above to select representative sample points. In the model training and testing phase, the GPU parallel random forest method developed in this study was employed. For the result analysis, we evaluated the proposed method’s performance in terms of micro-terrain classification accuracy and efficiency. Finally, we examined the model’s applicability and limitations based on the analysis outcomes.

While the methods employed throughout the experimental process are based on the data from this study, the modifications and innovations incorporated into these methods enhance their scalability.

### The performance evaluation of micro-terrain detection based on parallel random forest model

To assess the model’s performance, evaluation metrics, including the confusion matrix, accuracy, kappa coefficient, and others, were employed. The confusion matrix, a two-dimensional matrix, illustrates the disparities between the actual and predicted outcomes of the test set, providing an intuitive assessment of classification performance. Accuracy, widely employed, serves as a metric to assess the overall classification performance. The kappa coefficient, considering all aspects of the confusion matrix, provides an evaluation of the model’s overall classification performance^34^. Commonly used single-class evaluation metrics encompass precision and recall^35^.

Table 5 displays the confusion matrix of the test set, enabling the following conclusions to be drawn: (1) Among all micro-terrains categories, uplift exhibits the highest misclassification rate, with misclassified sample points distributed across all micro-terrain types, attributed to uplift being the most prevalent among these five types. (2) Six out of 2,480 saddle sample points are misclassified as canyons, while only two out of 2,453 canyon sample points are classified as saddles, validating the effectiveness of the optimized method in distinguishing between the saddle and the canyon. (3) The classification accuracy and kappa coefficient reached 97.96% and 0.974, respectively, indicating excellent classification performance of the model.

**Table 5:** Test set classification confusion matrix.

Micro-terrain categories	Uplift	Saddle	Canyon	Alpine watershed	Atypical type	Total
Uplift	2269	42	29	48	4	2392
Saddle	26	2438	6	1	9	2480
Canyon	31	2	2420	0	0	2453
Alpine watershed	41	0	0	2429	2	2472
Atypical type	1	9	0	0	2481	2491
Accuracy = 97.96%				Kappa coefficient = 0.974		

Table 6 presents the classification performance of different types of micro-terrain. Analysis of Table 6 reveals varying detection outcomes of the model across different micro-terrain types. Uplift demonstrates slightly inferior detection performance, with precision and recall rate of 95.82% and 94.86%, respectively. Conversely, the precision and recall of the remaining three types of typical micro-terrain exceed 97.50%.

**Table 6:** Classification performance evaluation of each category.

Categories	Precision	Recall
Uplift	95.82%	94.86%
Saddle	97.87%	98.31%
Canyon	98.57%	98.65%
Alpine watershed	98.02%	98.26%
Atypical type	99.40%	99.60%

### Experimental results of micro-terrain detection and discussion

**Fig. 8:**
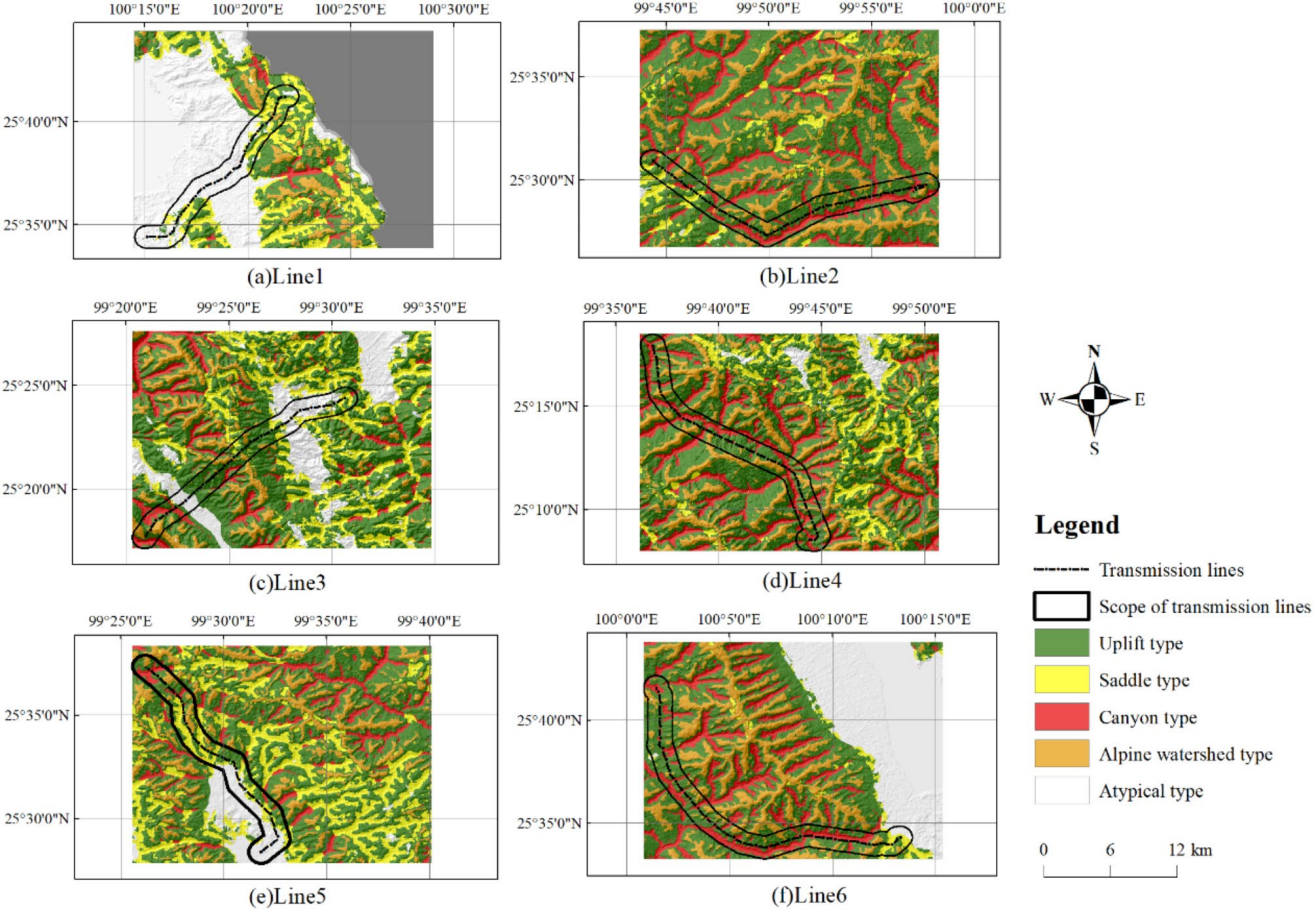
Detection results of micro-terrain.

To assess the classification effectiveness of the parallel random forest model, six transmission lines traversing diverse types of micro-terrain were selected from the study area. The micro-terrain detection range encompasses a 1-kilometer area on both sides of the transmission lines’ centerline. Figure 8 depicts the micro-terrain detection outcomes.

Table 7 presents the statistical analysis of micro-terrain detection results for the aforementioned transmission lines. Analysis of Table 7 reveals that Line 1 is predominantly characterized by atypical type of micro-terrain. Upon considering pertinent ground data, we ascertain that Line 1 traverses numerous residential areas. Conversely, the remaining five lines are predominantly dominated by uplift, primarily situated in mountainous regions. Furthermore, apart from uplift, the breakdown of these five lines is as follows: Line 2, Line 4, and Line 6 are primarily characterized by canyons and alpine watersheds; Line 3 encompasses various types of micro-terrain; Line 5 is chiefly composed of saddles and atypical type micro-terrain.

In the previous section, we noted that the precision of uplift type is relatively low. As shown in Table 7, this can be attributed to its wide distribution. Although the dataset used in this study includes a substantial number of sample points for the uplift type, including additional representative sample points in future research could further enhance classification accuracy.

**Table 7:** Statistics of micro-terrain detection results around transmission lines.

Line number	Uplift	Saddle	Canyon	Alpine watershed	Atypical type
Line 1	9,368	9,200	1	457	27,047
Line 2	36,265	2,772	16,875	7,410	10
Line 3	24,788	4,720	5,525	4,674	12,909
Line 4	35,187	14	16,208	8,970	0
Line 5	19,776	11,640	3,869	4,565	14,013
Line 6	41,299	2,467	19,380	9,109	1,765

**Fig. 9:**
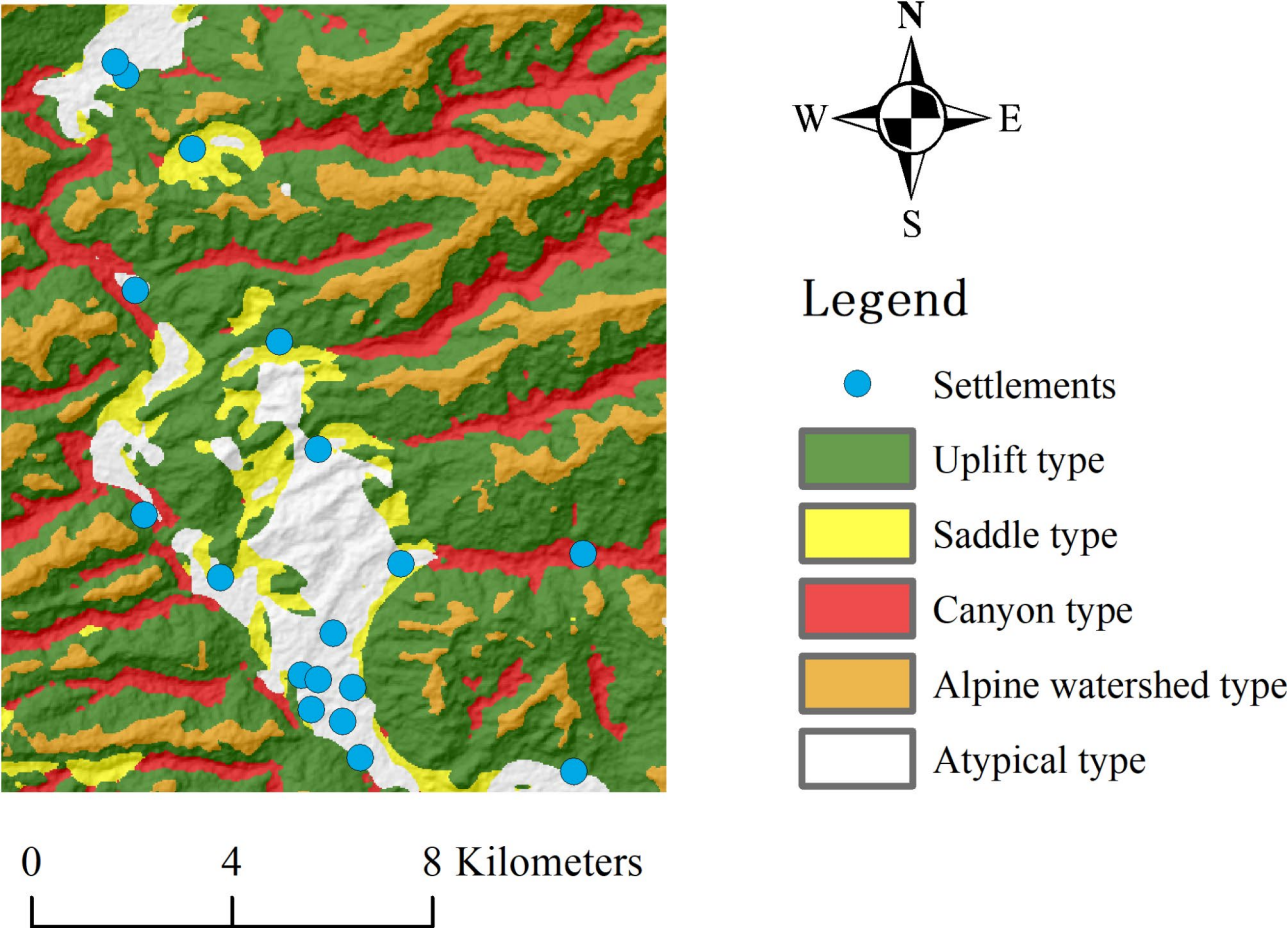
Comparison of distribution between settlements and atypical micro-terrain.

The atypical micro-terrain in this study refers to background features, such as settlements. To verify the stable applicability of the atypical micro-terrain type proposed in this paper, we overlaid the extracted atypical micro-terrain from the study area with the settlement distribution and compared their distributions for consistency. The results are shown in Fig. 9. The settlement distribution marked with blue dots shows a high degree of consistency with the extracted atypical micro-terrain. This demonstrates the effectiveness of the atypical micro-terrain type in background extraction and provides a reliable reference for its applicability in different regions.

### Comparative analysis of experimental results

#### Comparative analysis with the results of traditional methods

To validate the effectiveness of the proposed method in improving the accuracy of micro-terrain extraction, we compared it with the traditional method^17^. The results indicate that the traditional approach performs less effectively in the study area, achieving an accuracy of 75.19% and a Kappa coefficient of 0.642. A detailed comparison reveals that the traditional method is hard to distinguish saddles from canyons, resulting in a precision of only 46.09% for saddle extraction. In contrast, the proposed method significantly improves this precision to 97.87%. The reason for this gap is that traditional methods rely on rule-based knowledge, lack scalability, and are difficult to scale or extrapolate.

**Fig. 10:**
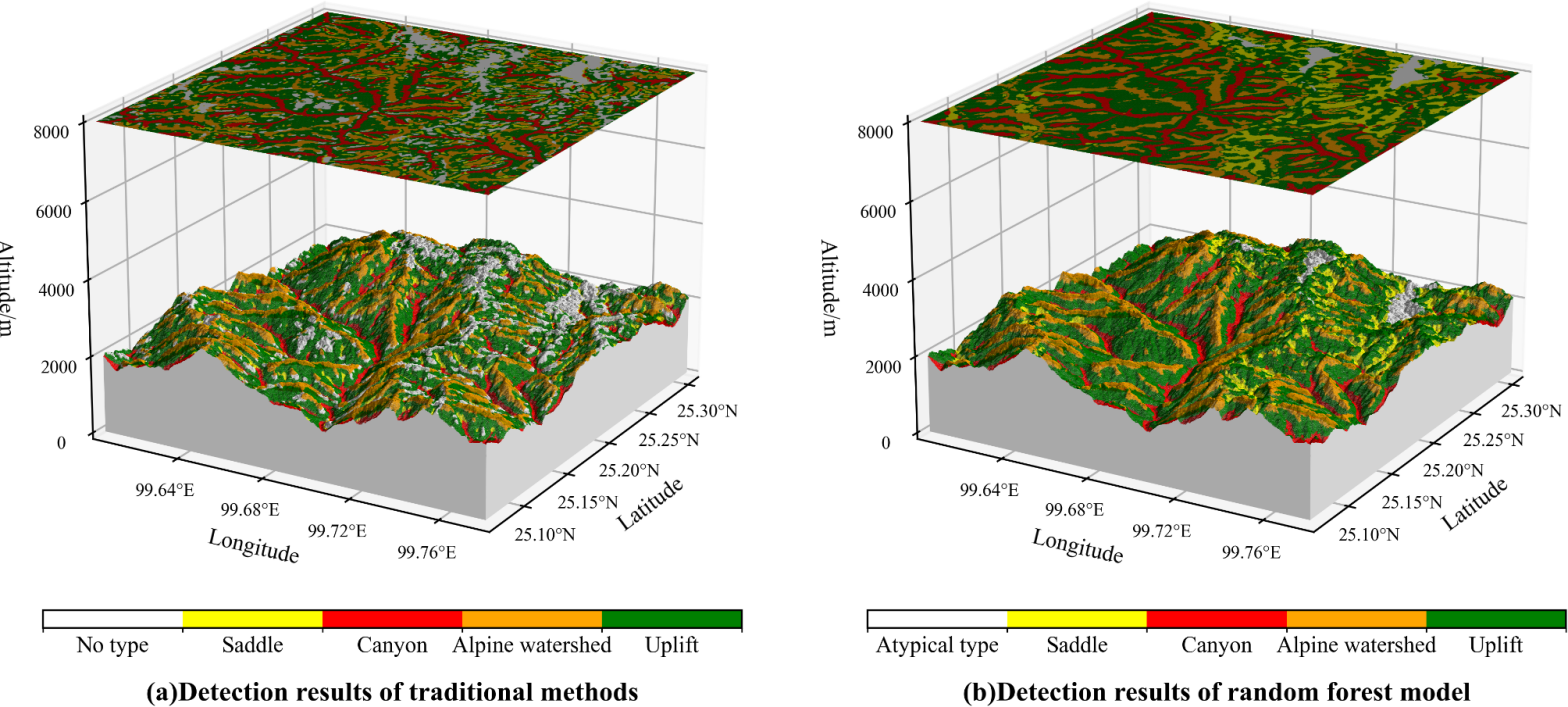
Comparison of results of Line 4.

Furthermore, the method proposed in this paper also solves the problem of incomplete classification. To illustrate this, Line 4 is used as a case study. In the case of Line 4, the traditional method directly extracts micro-terrain using a manually defined classification decision table. This traditional method, based on overlay analysis, may result in some grid points remaining unclassified, leading to incomplete classifications and disrupting the integrity of micro-terrain representation, as depicted in Fig. 10a. To address this issue, Sect. "Micro-terrain categories" introduces the concept of “atypical micro-terrain type” to facilitate the rational classification of unclassified points. Utilizing the parallel random forest model for micro-terrain detection, unclassified points are segmented into typical micro-terrain types and atypical micro-terrain type, effectively addressing detection incompleteness and ensuring the validity of classification, as illustrated in Fig. 10b. The final experimental results indicate that, when extracting the micro-terrain of Line 4, 13.04% of the grid points remain unclassified using the traditional method. In contrast, the improved method effectively addresses this issue by reasonably reassigning the unclassified points, ensuring complete classification.

To assess whether the difference in classification performance between the proposed method and the traditional method is statistically significant, we selected some additional data around the study area and created 10 datasets. These datasets were then classified using both traditional method and the method proposed in this study. The results indicate that the average classification accuracy of the traditional method across these 10 datasets is 67.71%, compared to 96.76% achieved by the proposed method. A paired t-test was subsequently conducted based on the results to determine whether the difference in classification performance between the two methods was statistically significant. Accuracy was used as the statistical indicator for analysis. The results showed a t-test statistic of 7.91 and a p-value below 0.05, indicating that the difference in classification performance is statistically significant.

#### Comparative analysis of the detection results of the saddle and canyon

**Fig. 11:**
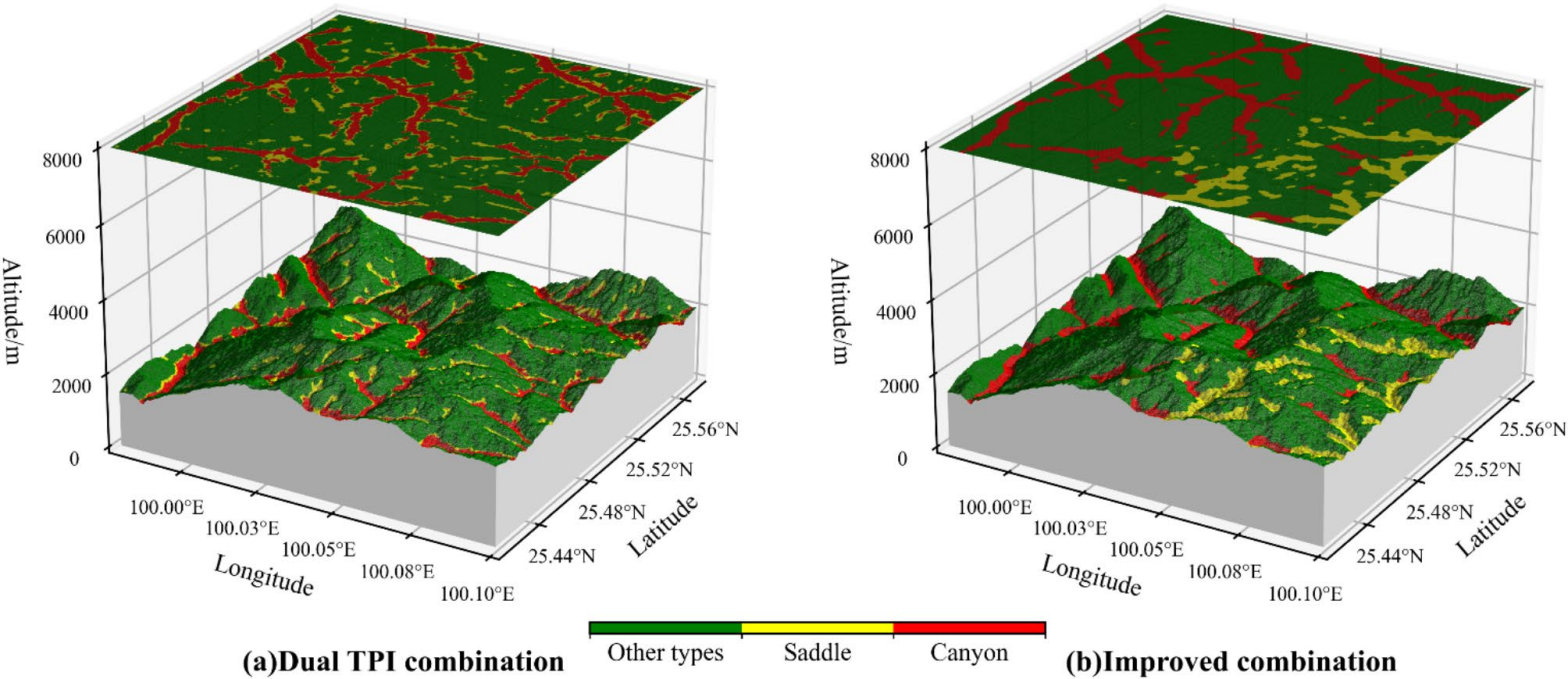
Detect saddle and canyon micro-terrain with different combinations.

In the previous section, we highlighted that one of the challenges with traditional methods is their inability to effectively distinguish between canyons and saddles. The traditional method^17^ relies on the “dual TPI combination” to differentiate between the two types (Fig. 11a): the saddle cannot effectively be detected, and part of the saddles are attached to the edges of the canyon.

This study optimizes the combination of characteristic factors, leveraging the enhanced combination outlined in Table 3 to delineate sample points for the saddle and the canyon. The micro-terrain outcomes are shown in Fig. 11b. Analysis of Fig. 11b reveals that the optimized combination strategy not only effectively identifies the saddle but also resolves the issue of saddle points adhering to the canyon edge.

### Experimental analysis of parallelized micro-terrain detection

CUML (CUDA Machine Learning) is a machine learning library based on the CUDA architecture. As an integral component of the RAPIDS (Real-time Acceleration Platform for Integrated Data Science) system, CUML accelerates machine learning tasks through GPU utilization^36,37^.

**Fig. 12:**
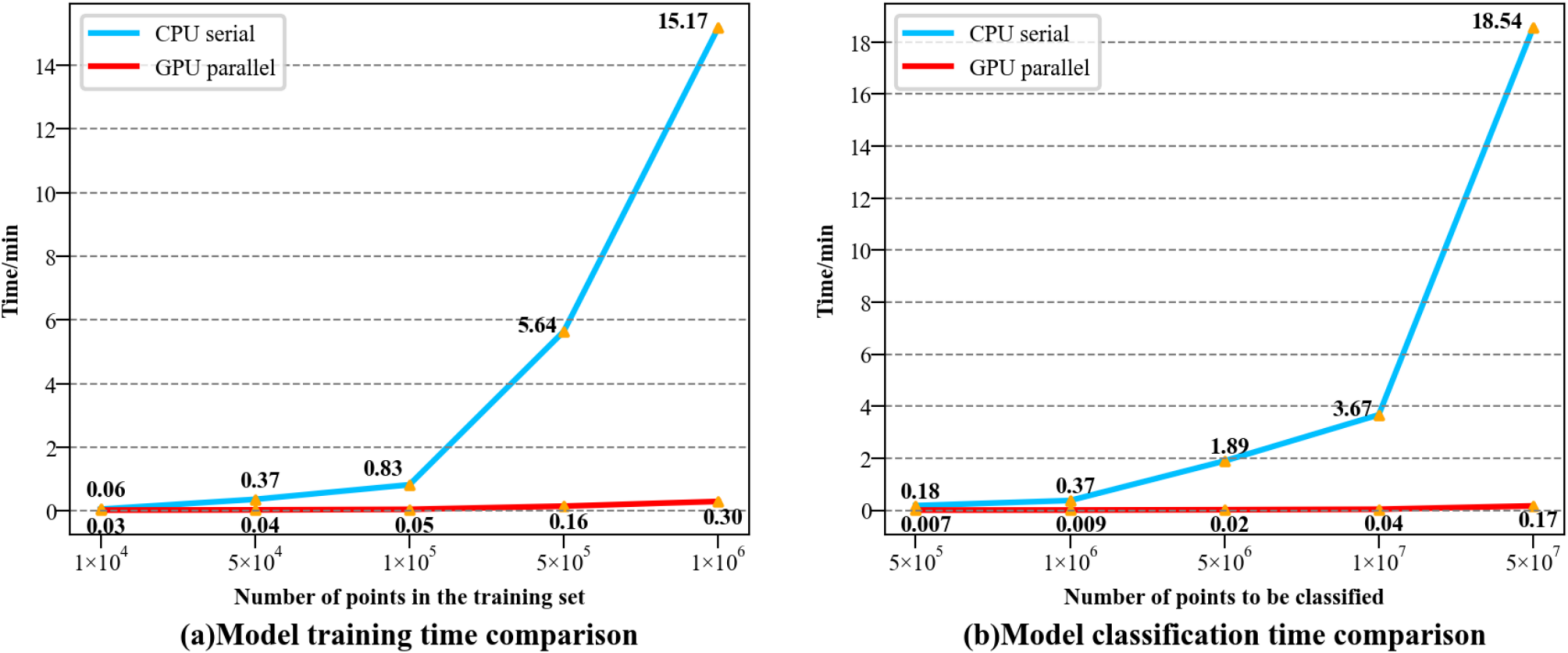
Time consumption comparison between CPU serial and GPU parallel model.

To assess the acceleration effect of parallel model training, a specific number of sample points are selected sequentially from the expanded sample dataset. Subsequently, the random forest model undergoes training using both CPU serial and GPU parallel methods. The training time comparison is depicted in Fig. 12a. As the number of sample points rises from 1 × 10^4^ to 1 × 10^6^, the training time for the CPU serial method escalates from 0.06 min to 15.17 min. In contrast, employing the GPU parallel method markedly reduces training time, with the acceleration ratio reaching 50.57 when the number of sample points is 1 × 10^6^. This observation underscores the pronounced training acceleration effect of the GPU parallel method, particularly evident when the training set size is large.

To validate the acceleration effect of parallel model classification, a substantial number of points are chosen for classification from 49 transmission lines, which span a total length of 1224.53 km. The micro-terrain around the transmission lines is detected using the random forest model, employing both the CPU serial and GPU parallel methods. The classification time comparison, illustrated in Fig. 12b, was obtained. Employing the CPU serial method, predicting 5 × 10^5^ points requires 0.18 min, while predicting 5 × 10^7^ points increases to 18.54 min. Conversely, the GPU parallel method significantly reduces classification time, manifesting a clear acceleration effect. Despite an increase in the number of points, the consumed time does not exhibit a notable upward trend. Merely 0.17 min are needed to predict 5 × 10^7^ points, with an acceleration ratio of 109.06 compared to the CPU serial method.

GPU parallel processing technology provides significant acceleration in both model training and prediction stages, ensuring that processing time does not increase substantially with data growth. The accelerated advantage of GPU facilitates rapid and efficient model processing when the study area spans a larger geographical area.

To further assess the practicality of the proposed method, we compared the energy consumption and computational resource requirements of the CPU serial and GPU parallel methods to evaluate their cost-effectiveness in practical applications. In the experiment, the maximum power consumption of the GPU used for parallel model training was 140 W, while the CPU used for serial model training had a maximum power consumption of 45 W. Despite the GPU’s power being several times that of the CPU, the GPU parallel method’s exceptionally high acceleration ratio results in significantly lower overall energy consumption compared to the CPU method. While this comparison is preliminary, it suggests that the proposed method contributes to reducing resource costs, especially when dealing with large datasets.

In conclusion, compared to the traditional CPU serial computing approach, the GPU parallel computing offers substantial advantages in terms of reducing both time and resource costs when processing large-scale datasets or complex terrain data. With enhanced hardware support, these benefits could be further amplified, thereby improving the real-time and dynamic response capabilities of terrain analysis. This potential for real-time data processing enables the acquisition of accurate dynamic terrain analysis and monitoring results, providing timely and reliable support for decision-making.

## Conclusions and discussion

This paper optimizes the calculation of characteristic factors and the strategy for micro-terrain detection using DEM raster data. We propose a detection method for micro-terrain around transmission lines based on optimized strategy of micro-terrain detection and a GPU parallel random forest model, successfully detecting micro-terrains on multiple transmission lines. The main research results of the article are as follows:

1. Our proposed method demonstrates reliable accuracy and robust applicability for detecting micro-terrain around transmission lines. It effectively addresses the detection incompleteness inherent in traditional methods, ensuring the classification of all raster points into their respective micro-terrain types. Compared to the traditional method, which achieved an accuracy of 75.19% and a Kappa coefficient of 0.642, the random forest-based method proposed in this study demonstrated significant improvement, achieving an accuracy of 97.96% and a Kappa coefficient of 0.974.
2. Optimizing the calculation and combination of characteristic factors enhances the detection performance of micro-terrain around transmission lines. The introduction of profile curvature and elevation coefficient of variation facilitates effective differentiation between saddle and canyon, thereby clarifying the classification boundary. The saddle recognition precision of the traditional method is below 50%, whereas the method proposed in this study increases it to 97.87%, demonstrating substantial improvement.
3. The efficiency of the GPU parallel random forest model in training and classification significantly surpasses that of the CPU serial method. As the micro-terrain area expands, the GPU parallel method achieves an acceleration ratio of 109.06 compared to the CPU serial method for completing the same classification task.

Micro-terrain detection informs the layout of transmission lines, enabling the avoidance of areas with complex terrain and disaster susceptibility, thereby enhancing the reliability and stability of power transportation. Analyzing micro-terrain during transmission line operation assesses risk and facilitates the formulation of effective maintenance strategies. While the research presented in this paper is grounded in a theoretical framework, future collaboration with stakeholders, such as power grid companies, and conducting practical experiments could more effectively validate the real-world impact of the findings.

While the model proposed in this research demonstrates high classification accuracy and efficiency, it may encounter several potential challenges and limitations in practical applications.

Firstly, while the GPU-accelerated parallel random forest model employed in this study demonstrates considerable efficiency improvements on current hardware configurations and datasets, its performance under different situations remains to be further validated. The model’s performance may vary under different hardware configurations, including various types of GPUs or CPUs, or even in distributed computing environments. Future research will focus on evaluating the model’s scalability with larger datasets and investigating the effects of diverse hardware configurations on its performance.

Secondly, the quality of the input data has a direct influence on the model’s performance. The dataset employed in this study is relatively limited, which may affect the model’s scalability. A more comprehensive dataset will be required in future research to facilitate further model development. In regions characterized by complex terrain and dense vegetation, acquiring high-quality raster DEM data can be challenging, thereby hindering the accurate extraction of micro-terrain features. Therefore, future research should prioritize the diversification of data sources through the integration of remote sensing images, LiDAR, and other relevant datasets to improve the reliability of the input data. Although integrating diverse data sources holds significant potential for enhancing the reliability and accuracy, it also presents challenges related to data alignment, calibration, and processing. For instance, remote sensing images may suffer from issues such as resolution limitations and atmospheric interference, whereas LiDAR data is prone to errors, particularly in densely forested regions. To address these challenges, future research could explore advanced data fusion techniques, such as multi-source image registration, machine learning-based alignment methods, and advanced filtering techniques, to enhance data quality and consistency.

Thirdly, current research primarily focuses on extracting specific micro-terrain types; however, the model’s adaptability may be constrained when confronted with a broader range of terrain types and evolving application scenarios. To address this limitation, future research could investigate more flexible and scalable deep learning models, such as convolutional neural networks (CNNs) and semantic segmentation models^11,12^, and apply these to the field of micro-terrain recognition. Moreover, the scalability of the current model can be improved through the integration of multiple models. For instance, incorporating transfer learning techniques can improve the model’s adaptability to new scenarios. Related studies have demonstrated that random forests and transfer learning can be effectively combined^38^. Future research could also explore the integration of traditional machine learning methods (such as random forests) with CNNs to develop a hybrid model, thereby leveraging the advantages of both approaches and improving the accuracy of complex terrain classification tasks^39^.

Although this study primarily focuses on detecting micro-terrain around transmission lines, the proposed method exhibits broad applicability in other fields requiring micro-terrain analysis. In urban planning, it can be employed to analyze terrain features such as slopes, depressions, and ridges, thereby aiding in the design of sustainable infrastructure, optimizing land use, and mitigating risks associated with natural disasters, such as flooding and landslides. In forestry, the method can identify areas susceptible to soil erosion, waterlogging, thus supporting forest management and conservation efforts. In hydro-geomorphological hazard studies, this approach improves flood risk assessments, enhances landslide predictions, and strengthens disaster mitigation strategies by accurately classifying terrain features such as watersheds, valleys, and uplifts.

Looking ahead, integrating engineering, environmental science, and geospatial analysis will offer more comprehensive solutions for micro-terrain detection, improve the accuracy of terrain analysis and fully consider ecological and social impacts. This interdisciplinary approach can effectively bridge technological advancements with practical applications, fostering more sustainable and effective strategies for land use, environmental protection, and disaster risk management.

## Data Availability

The data that support the findings of this study are available on request from the corresponding author upon request.

## References

[1] Tan, X. et al. Power supply and demand balance during the 14th five-year plan period under the goal of carbon emission peak and carbon neutrality. *Electr. Power*. **54** (05), 1–6 (2021).

[2] Huang, X. B. & Cao, W. Review of the disaster mechanism of transmission lines. *J. Xi’an Polytech. Univ.* **31** (5), 589–605 (2017).

[3] Hu, Y., Liu, K., Wu, T. & Su, Z. M. Analysis of influential factors on Operation Safety of Transmission Line and countermeasures. *High. Volt. Eng.* **40** (11), 3491–3499 (2014).

[4] Goel, A. Design of transmission lines for atmospheric icing. In Atmospheric Icing of Power Networks. Springer Netherlands, 327-371. (2008).

[5] Zhiguo, H. et al. Review on disaster of Wire Icing in China. *J. Appl. Meteorol. Sci.* **32** (5), 513–529 (2021).

[6] Wang, Y. W. & Qin, C. Z. Review of methods for landform automatic classification. *Geogr. Geo-Inf. Sci.* **33** (4), 16–21 (2017).

[7] Minár, J. & Evans, I. S. Elementary forms for land surface segmentation: the theoretical basis of terrain analysis and geomorphological mapping. *Geomorphology* **95** (3–4), 236–259 (2008).

[8] Jasiewicz, J. & Stepinski, T. F. Geomorphons—a pattern recognition approach to classification and mapping of landforms. *Geomorphology* **182**, 147–156 (2013).

[9] Hengl, T. & Rossiter, D. G. Supervised landform classification to Enhance and replace photo-interpretation in Semi‐detailed soil survey. *Soil Sci. Soc. Am. J.* **67** (6), 1810–1822 (2003).

[10] Tian, D., Liu, A. L., Ding, H., Zhang, W. & Qi, W. Improvement of object-oriented classification method for landform types. *Geogr. Geo-Inf. Sci.* **32** (2), 46–50 (2016).

[11] Yang, J. et al. Deep learning-based automated terrain classification using high-resolution DEM data. *Int. J. Appl. Earth Obs. Geoinf.* **118**, 103249 (2023).

[12] Ouyang, S. et al. A fine-grained genetic landform classification network based on multimodal feature extraction and regional geological context. *IEEE Trans. Geosci. Remote Sens.* **60**, 1–14 (2022).

[13] Koval, D. O. & Chowdhury, A. A. An investigation into extreme-weather-caused transmission line unavailability. In *IEEE Power Engineering Society General Meeting**IEEE* 2425–2428. (2005).

[14] Zhang, X. Mechanism and countermeasures for ice-coated transmission line in micro-terrain and microclimate region. *Power Syst. Technol.* **31** (2), 87–89 (2007).

[15] Drăguţ, L. & Eisank, C. Automated object-based classification of topography from SRTM data. *Geomorphology* **141**, 21–33 (2012). 22485060 10.1016/j.geomorph.2011.12.001 PMC3312788

[16] Jing, H., Ying, D. & Xingliang, J. Feature extraction and identification method of ice-covered saddle mircotopography for transmission lines. *Electr. Power*. **55** (8), 135–142 (2022).

[17] Zhou, F., Meng, F., Zou, L., Li, Z. & Wang, J. Automatic extraction of Digital Micro Landform for Transmission Lines. *Geomatics Inform. Sci. Wuhan Univ.* **47** (9), 1398–1405 (2022).

[18] Dornik, A., DRĂGUŢ, L. & Urdea, P. Classification of soil types using geographic object-based image analysis and random forests. *Pedosphere* **28** (6), 913–925 (2018).

[19] Wang, L., Ma, F. H. & Wu, W. Fuzzy slope position segmentation based on random forest. *J. Southwest. China Normal Univ. (Natural Sci. Edition)*. **43** (01), 10–17 (2018).

[20] Zhou, F., Zou, L., Liu, X. & Meng, F. Micro landform classification method of grid DEM based on convolutional neural network. *Geomatics Inform. Sci. Wuhan Univ.* **46** (8), 1186–1193 (2021).

[21] Chen, L., Zhao, L. & Li, K. The research of parallel terrain factor Algorithm based on CUDA. *Beijing Surv. Mapp.* (04), 9–12 (2017).

[22] Zeng, Y. *Identification Method of Typical Micro-terrain and Micro-climate in Ice-covered Area of Transmission Line* (Chongqing University, 2022).

[23] Weiss, A. Topographic position and landforms analysis. In *Poster Presentation, ESRI User Conference*, San Diego, CA. (2001).

[24] Li, T. W., Liu, X. J. & Tang, G. A. Influence of terrain complexity on slope and aspect. *J. Mt. Sci.* **22** (3), 272–277 (2004).

[25] Chen, J. *Study on Weighted Terrain Position Index Its Application* (Nanjing Normal University, 2019).

[26] Cao, W. H., Tao, H. P., Kong, B., Liu, B. T. & Sun, Y. L. Automatic identification of geomorphology partition of Southwest China based on DEM data. *Soil. Water Conserv. China*. **3**, 38–41 (2011).

[27] Guo, Z. Z., Yin, K. L., Huang, F. M., Fu, S. & Zhang, W. Evaluation of landslide susceptibility based on landslide classification and weighted frequency ratio model. *Chin. J. Rock Mechan. Eng.* **38** (2), 287–300 (2019).

[28] Yang, Q. K. et al. Application of DEMs in regional soil erosion modeling. *Geomatics World*. **7** (1), 25–32 (2009).

[29] Wilson, J. P. & Gallant, J. C. (eds) *Terrain Analysis: Principles and Applications* (Wiley, 2000).

[30] Fang, K. N., Wu, J. B., Zhu, J. P. & Xie, B. C. A review of technologies on Random forests. *J. Stat. Inform.* **26** (03), 32–38 (2011).

[31] Dong, L., Ge, W. C. & Chen, K. L. Study on application of parallel computation on CUDA. *Inform. Technol.* **34** (04), 11–15 (2010).

[32] Grahn, H., Lavesson, N., Lapajne, M. H. & Slat, D. CudaRF: a CUDA-based implementation of random forests. In *9th IEEE/ACS International Conference on Computer Systems and Applications (AICCSA)* IEEE, 95–101. (2011).

[33] Kaur, K., Sagar, A. K. & Chakraborty, S. Accelerating the performance of sequence alignment using machine learning with RAPIDS enabled GPU. *J. Curr. Sci. Technol.* **12** (3), 462–481 (2022).

[34] Dongyang, X., Lin, Z. & Guoqing, L. MaxEnt-based multi-class classification of land use in remote sensing image interpretation. *Remote Sens. Nat. Resour.* **35**(2) (2023).

[35] Xu, L. L. & Chi, D. X. Machine learning classification strategy for imbalanced datasets. *Comput. Eng. Appl.* **56** (24), 12–27 (2020).

[36] Raschka, S., Patterson, J. & Nolet, C. Machine learning in python: main developments and technology trends in data science, machine learning, and artificial intelligence. *Information* **11** (4), 193 (2020).

[37] Jaykar, S., Kokate, T., Joshi, A. D. & Sawant, S. T. GPU Accelerated multiple regression using cuML. *Jaypee Univ. Eng. Technol. Raghogarh Guna (MP)* 13 (2020).

[38] Li, D. *Research of Random Forest Transfer Learning Based on Instance* (Shanxi Normal University, 2018).

[39] Nijhawan, R., Das, J. & Balasubramanian, R. A hybrid CNN + random forest approach to delineate debris covered glaciers using deep features. *J. Indian Soc. Remote Sens.* **46**, 981–989 (2018).

